# Procedural sedation and analgesia practices by emergency physicians in the Netherlands: a nationwide survey

**DOI:** 10.1186/s12245-017-0159-2

**Published:** 2017-12-15

**Authors:** Maybritt I. Kuypers, Gaël J. P. Smits, Suzanne C. Valkenet, Wendy A. M. H. Thijssen, Frans B. Plötz

**Affiliations:** 1 Academic Medical Centre/ University of Amsterdam, Amsterdam, The Netherlands; 2 Emergency Department, Catharina Hospital, Eindhoven, The Netherlands; 3 VU University Medical Center, Amsterdam, The Netherlands; 4 Emergency Department, Catharina Hospital, Eindhoven, The Netherlands; 5 Department of Pediatrics, Tergooi Hospital, Blaricum, The Netherlands; 6 Tergooi Hosptial, Hilversum, The Netherlands

**Keywords:** Sedation, Analgesia, Emergency medicine, Training, Paediatric and adult

## Abstract

**Background:**

Several efforts have been made to assure and to improve the quality of procedural sedation and analgesia (PSA) performed by emergency physicians (EPs) in The Netherlands. This study investigated the current PSA practice and competences of EPs in both adult and paediatric patients. In particular, if residency and current training, awareness of guidelines is sufficient for registered EPs to adequately perform PSA and if the availability of both adult and paediatric PSA in the ED is adequate.

**Methods:**

A cross-sectional nationwide survey was performed amongst Dutch EPs (*n* = 463) in June 2016. We collected data on background, training, practice, and competencies of both adult and paediatric PSA. We investigated guideline adherence, reasons for not performing PSA, and desired improvements.

**Results:**

The respondents (*n* = 191) represented 84.6% hospitals with EPs and 41.3% of all EPs in The Netherlands. Nearly all EPs (97.8%) performed PSA in adult patients compared to only 59.1% who performed PSA in paediatric patients (*p* < 0.001). The major reason for not performing paediatric PSA was caused by a lack of exposure during the training-program (74.1%). PSA-guideline knowledge (98.3%) and PSA related adverse event registration (98.3%) were excellent. Lack of 24/7-availability of both adult and paediatric emergency department PSA was mainly caused by a shortage of EPs. Self-reflection indicated that EPs feel less competent in performing paediatric PSA when compared to adult PSA.

**Conclusion:**

This nationwide survey demonstrates that there is still a significant gap between the performance of adult and paediatric PSA even though guideline adherence and registration of PSA-related adverse events appear to be adequate. Enhancement of paediatric PSA training in combination with an increase of EP-staffing can help improve the availability of adult and paediatric PSA in the emergency department.

**Electronic supplementary material:**

The online version of this article (10.1186/s12245-017-0159-2) contains supplementary material, which is available to authorized users.

## Background

Procedural sedation and analgesia (PSA) is defined as a technique of administering sedatives or dissociative agents with or without analgesics to induce a state that allows the patient to tolerate unpleasant procedures while maintaining cardiorespiratory function. PSA is an integral part of emergency medicine (EM) practice and can be performed safely and effectively in adult and paediatric patients in the emergency department (ED) by trained emergency physicians (EPs) [1–5]. PSA delivered in the ED by EPs provides many advantages for both the patient and hospital, like reduced waiting times, reduced operating room and anaesthesia usage, reduced hospital admissions, and considerable savings [6].

In The Netherlands, the first 3-year EM training program commenced in 2000. PSA was introduced in 2005 by EP faculty from the USA [7]. Since then, multiple efforts have been made to assure and to improve the quality of PSA performed by EPs [8]. In 2012, a national guideline on PSA was implemented [9]. Simultaneously, the Netherlands Society of Emergency Physicians (NSEP) distributed a guideline on emergency PSA, including an updated standard data collection form, intended to aid proper registration and guideline adherence [10]. Organized registration of adverse events due to PSA was made mandatory and used as a quality marker. Following in 2012, NSEP developed a PSA training course and examination, mandatory for all EPs performing PSA in the ED. A survey amongst EM residents in 2013 showed that 45.7% deemed themselves competent in performing PSA [11]. Since December 2014, PSA has become fully integrated into the national EM curriculum, including a mandatory national PSA qualification examination and clinical evaluation of PSA in practice. It remains unclear, however, the effect of all these measures on current practice of PSA by registered EPs in The Netherlands. This is important because evaluation provides valuable feedback on the design and the implementation of the program.

The aim of this survey study was to investigate the current PSA practice and competences of Dutch EPs in both adult and paediatric patients. In particular, if residency and current training, awareness of guidelines is sufficient for registered EPs to adequately perform PSA and if the availability of both adult and paediatric PSA in the ED is adequate.

## Methods

### Study design and setting and participants

The STROBE statement was used in the design of this study [12]. Approval for this study was obtained through the Scientific Review Committee of the Tergooi Hospital. The committee granted further review board exemption. No patient data was included in this study.

Two authors created the survey (MK, SV). Thereafter, the scientific committee of NSEP piloted the survey. After approval of the survey, NSEP provided access to the list of all registered EPs. In June 2016, an online survey was distributed via email to all registered EPs. Only EPs currently working in a Dutch ED were included for participation. Participation was on a voluntary basis and confidentiality and anonymity were guaranteed to avoid potential participation bias. After 1 week, a reminder was sent to the non-responders.

### Variables and measurements

Data on general and demographic information, experience and practice of PSA in both adult and paediatric patients, residency and current PSA training, guideline use and indicators, and finally PSA-related competencies were collected by use of a digital questionnaire (Additional file 1). Briefly, questions regarding general and demographic data were open or multiple-choice. Most questions related to PSA training and to PSA practice were initially dichotomous and then followed by multiple-option answers. In order to quantify the performance rate, we choose for multiple-choice questions. Questions regarding the technical aspects of the PSA performance were evaluated using the national guideline as a reference standard. To measure the clinicians’ opinions or attitudes regarding the adult and paediatric PSA-related competencies, we used a five-point Likert scale to indicate the amount of agreement (strongly agree, agree, neutral, disagree, strongly disagree).

### Analysis

Statistical analysis was performed using IBM SPSS Statistics, Version 22.0. Armonk, NY: IBM Corp.

Group differences between PSA in adults and paediatric patients were analysed for nominal/ordinal variables by chi-square test and for continuous variables by one-sample *t* test or Mann-Whitney *U* test where appropriate. Multivariate linear regression was applied to examine the differences in PSA competencies in adults and in paediatric patients, adjusted for demographic variables. All tests were two-tailed. A *p* value of less than 0.05 was considered to be statistically significant.

## Results

### Participants

According to NSEP, in June 2016, there were 463 registered EPs in The Netherlands and the survey was accessed by 41.3% (191/463). Ten respondents did not complete the survey and were therefore excluded from further analysis, leaving 181 responses for analysis. In The Netherlands, there are 87 EDs, of which 65 have at least one EP working in their ED. The respondents represented 55/65 (84.6%) of the Dutch hospitals with EPs.

### Descriptive data

The majority of the respondents were female (65.7%). The mode for the respondents’ age group was 35–39 years (37.0%). A small majority had an EP-registration of less than 5 years (52.8%). All but one respondent completed their EM training in The Netherlands. The mode for clinical working hours per week was 25–36 h (73.5%).

### Current practice of adult versus paediatric PSA in the ED

The current practices of adult PSA, including indications for PSA and medication used are displayed in Table 1. PSA in adult emergency patients was performed by 97.8% (177/181) of the EPs, whereas in paediatric patients this was 59.1% (107/181) (*p* < 0.001). PSA was performed in the adult population with a frequency of at least once per week by 53.7% (95/177) of the EPs, and for the paediatric population, this frequency was reached by 19.6% (21/107). The performance rate for adult PSA was declared sufficient to maintain skill-competency by 75.7% (134/177), and for paediatric PSA, the rate was deemed sufficient by 54.2% (58/107). The most common reason for not performing PSA in adults was the absence of additional EPs in the ED to cover other ED patient care. The most common reason given for not performing paediatric PSA in the ED was lack of experience to perform paediatric PSA.

**Table 1:** PSA practice in adults versus paediatric ED patients in The Netherlands

	Adult		Children	
	*n*	(%)	*n*	(%)
Do you perform PSA procedures? Yes:	177	(97.8)	107	(59.1)*
Frequency PSA procedure (per individual EP)				
-Five times a week	4/177	(2.3)	–	
-Two-four times a week	36/177	(20.3)	8/107	(7.5)
-Once a week	*55/177*	*(31.1)*	13/107	(12.1)
-Once per 2 weeks	38/177	(21.4)	25/107	(23.4)
-Once per month	29/177	(16.4)	*39/107*	*(36.4)*
-< Once per month	15/177	(8.5)	22/107	(20.6)
Is this frequency enough to maintain your skills? YES:	134/177	(75.7)	58/107	(54.2)
Does your ED have 24/7 PSA availability? YES	56/181	(30.9)	32/181	(17.7)
Why does your ED not have 24/7 PSA availability?^a^				
-Physicians who perform paediatric PSA are not always available	–		53/149	(35.6)
-The ED is not staffed with EPs 24/7	*94/125*	*(75.2)*	*62/149*	*(41.6)*
-Despite 24/7 staffing with EPs, the EP is not always available	52/125	(41.6)	47/149	(31.5)
-Not all EPs in the ED are capable in performing PSA	11/125	(8.8)	36/149	(24.2)
-Other	24/125	(19.2)	47/149	(31.5)
First choice sedative				
-Propofol	*14/177*	*(83.1)*	31/107	(29.0)
-Midazolam	5/177	(2.9)	8/107	(7.5)
-Esketamine	20/177	(11.3)	*54/107*	*(50.5)*
-Etomidate	2/177	(1.1)	0	
-Nitrous oxide 50:50	1/177	(0.5)	13/107	(12.1)
-Other	2/177	(1.1)	1/107	(0.9)
First choice analgesic				
-Fentanyl	*153/177*	*(86.4)*	*52*	*(48.6)*
-Sufentanil	0		1	(0.9)
-Remifentanil	0		0	
-Morphine	1/177	(0.6)	0	
-Paracetamol	0		3	(2.8)
-NSAID	1/177	(0.6)	1	(0.9)
-Esketamine	20/177	(11.2)	36	(33.7)
-Nitrous oxide 50:50	0		3	(2.8)
-Local (for example: lidocaine)	0		2	(1.9)
-None	1/177	(0.6)	8	(7.5)
-Other	1/177	(0.6)	1	(0.9)
Use of intranasal route for analgesics/sedatives? YES:	88/177	(49.7)	95/107	(88.8)*
Indications^a^				
-Dislocation of the hip	*135/177*	*(76.3)*	0	
-Dislocation of the shoulder	77/177	(43.5)	1/107	(0.9)
-Dislocation of the elbow	21/177	(11.9)	6/107	(5.6)
-Other joint dislocations	11/177	(6.2)	*1/107*	*(0.9)*
-Dislocated fracture of the arm	25/177	(14.1)	*95/107*	*(88.8)*
-Dislocated fracture of the leg	19/177	(24.9)	19/107	(17.8)
-Incision of an abscess	23/177	(13.0)	2/107	(1.9)
-Cardioversion	36/177	(20.3)	0	
-Foreign body ENT	0		1/107	(0.9)
-Wound management face	2/177	(1.1)	18/107	(16.8)
-Other wound management	4/177	(2.3)	31/107	(45.8)
-CT scan	3/177	(1.7)	0	
-Chest tube	6/177	(3.4)	0	

^a^Multiple answers possible

*EM* emergency medicine, *PSA* procedural sedation and analgesia, *ED* emergency department, *CT* computed tomography, *ENT* ear nose throat

* *p* < 0.001

### PSA training during and after residency

The training received in paediatric PSA was significantly lower than PSA in adults (*p* < 0.05) (Table 2). Only 40 of 181 (22%) EPs received paediatric PSA training, and of those who received training, only 30.0% (12/40) believed it to be sufficient (Table 1). Lack of significant exposure to due to the short duration of the 3-year residency-training program was considered one of the major causes for this insufficiency. The PSA training provided by NSEP had been attended by 46.4% of the respondents. The desire to continue vocational training was expressed by 50.3%. The vocational training with the highest demand was paediatric PSA training 74.7% (68/91).

**Table 2:** Survey on adult and paediatric PSA training within the Dutch EM training program

	Adults		Children	
	*n*	(%)	*n*	(%)
Were you trained in PSA during your EM training program? YES:	112/181	(61.9)	40/181	(22.1)*
Was the PSA training during your EM training program sufficient? YES:	46/112	(41.1)	12/40	(30.0)
What was the reason for this insufficient training?^a^				
-Lack of exposure due to a short (3 years) EM training program	28/66	(42.4)	20/28	(71.4)
-Lack of exposure due to competition with other specialties	4/66	(6.1)	1/28	(3.6)
-Insufficient training because mentors did not perform PSA	20/66	(30.3)	8/28	(28.6)
-Insufficient airway management skills training	4/66	(6.1)	2/28	(7.1)

^a^Multiple answers possible

*EM* emergency medicine, *PSA* procedural sedation and analgesia

* *p* < 0.05

### Quality: guideline use and quality indicators

Almost all EPs 98.3% have knowledge of the national guideline on PSA and frequently use it as their default protocol (81.5%). The NSEP practical emergency PSA guideline was used slightly more frequently 87.5% (147/168). PSA procedures were registered by 99.4% either on paper (61.3%) or in an electronic patient record (38.1%). PSA-related adverse event registration, in accordance with the quality standard set by the NSEP, was met according to 98.3%. First choices of PSA sedatives and analgesics are displayed in Table 1.

### Self-reflection on competencies regarding PSA

EPs reflected on their PSA competencies in both adult and paediatric PSA (Tables 3 and 4). The EPs were queried on their capabilities and competencies in performing PSA and if they were able to perform advanced life support (ALS)/advanced paediatric life support (APLS) appropriately. They agreed significantly stronger (*p* < 0.001) when reflecting their PSA competencies in adults compared to paediatric patients. This effect was not altered by age, years of registration, or work experience of the EP.

**Table 3:** Self-reflection of emergency physicians on adult PSA in The Netherlands (*n* = 177)

	Strongly agree		Agree		Neutral		Disagree		Strongly disagree	
	*n*	(%)	*n*	(%)	*n*	(%)	*n*	(%)	*n*	(%)
I am capable and competent in performing PSA in adults	101	(57.1)	70	(39.5)	4	(2.3)	2	(1.1)	0	(0.0)
I am able to perform ALS appropriately	151	(85.3)	26	(14.7)	0	(0.0)	0	(0.0)	0	(0.0)
I am aware of the (contra-)indications and precautions for PSA	115	(65.0)	61	(34.5)	1	(0.5)	0	(0.0)	0	(0.0)
I am able to recognize and treat the most common adverse events and/or complications	102	(57.6)	72	(40.7)	3	(1.7)	0	(0.0)	0	(0.0)

*ALS* Advanced life support, *PSA* procedural sedation and analgesia

**Table 4:** Self-reflection of emergency physicians on paediatric PSA in The Netherlands (*n* = 107)

	Strongly Ag agree		Agree		Neutral		Disagree		Strongly disagree	
	*n*	(%)	*n*	(%)	*n*	(%)	*n*	(%)	*n*	(%)
I am capable and competent in performing paediatric PSA	33	(30.9)	61	(57.0)	10	(9.3)	3	(2.8)	0	(0.0)
I am able to perform APLS appropriately	60	(56.1)	46	(43.0)	1	(0.9)	0	(0.0)	0	(0.0)
I am able to inform the patient, parents and/or a legal guardian about the sedation technique, the effects, possible adverse events and alternatives	59	(55.2)	47	(43.9)	1	(0.9)	0	(0.0)	0	(0.0)
I can guarantee paediatric friendly circumstances before, during and after the procedure	32	(30.0)	57	(53.2)	17	(15.9)	1	(0.9)	0	(0.0)
I am able to effectively use local or topical anaesthesia when needed	55	(51.4)	48	(44.9)	4	(3.7)	0	(0.0)	0	(0.0)
The age of the patient determines if I perform PSA or not	19	(17.8)	54	(50.4)	11	(10.3)	20	(18.7)	3	(2.8)
I only apply PSA in paediatric patients in the direct presence of another specialist	0	(0.0)	7	(6.5)	9	(8.4)	52	(48.6)	39	(36.5)
I only apply PSA in paediatric patients after consulting an anaesthesiologist/paediatrician.	0	(0.0)	2	(1.9)	4	(3.7)	58	(54.2)	43	(40.2)

*APLS* advanced paediatric life support, *PSA* procedural sedation and analgesia

## Discussion

The present study provides insight into the current PSA practice and competences of Dutch EPs in both adult and paediatric patients. First, nearly all EPs (97.8%) performed PSA in adult patients, compared to only 59.1% who performed PSA in paediatric patients. Second, self-reflection indicated that EPs felt less competent in performing paediatric PSA when compared to adult PSA. Consequently, there is a significant gap between adult and paediatric PSA performance by EPs in Dutch EDs. The most common reason for not performing paediatric PSA according to the EPs was the lack of exposure due to the relatively short 3-year training program.

Other countries report similar gaps between adult and paediatric PSA in the ED [13, 14]. McCoy et al. addressed the challenges of practice and provision of paediatric PSA in the UK and Ireland. They concluded that among others that lack of formal training and a lack of recognition of PSA as a specialised EM skill contributed to the difficulties to provide paediatric PSA in the ED [13]. The unavailability of PSA around-the-clock for both the adult and paediatric populations in Dutch EDs is another concern. As shown in the results, EPs appear to be the foremost providers of PSA in the ED for both adult and paediatric patients. Currently, only 20.7% of the 87 EDs have 24/7-coverage by EPs. Increase of EP staff could therefore may be an effective step in reaching good quality emergency PSA service for all ED patients at all times. An additional positive side-effect may be further reduction of healthcare costs as ED sedation has shown to offer considerable savings, compared with theater-based management [6].

The second aim was to investigate the competencies of Dutch EPs in both adult and paediatric PSA. To our knowledge, this is the first cross-sectional study on a national level amongst EPs demonstrating their self-reflection on PSA-related skills. We would like to stress that because there is a lack in confidence to perform paediatric PSA, this does not automatically imply that the PSA provided is of a lesser quality. Multiple Dutch studies have shown that EPs can perform safe and effective PSA, and they have similar adverse event rates when compared their international counterparts [5, 7, 15]. However, this lack in confidence does result in fewer EPs performing paediatric sedations in the ED, resulting in less skill maintenance and less exposure for the EPs in training. Moreover, this causes a potential serious risk for under treatment of pain in the paediatric ED population.

According to the respondents, the root cause of this observed difference between adult and paediatric PSA appears to be due to the lack of exposure of paediatric PSA during the relatively short 3-year training program in The Netherlands. A 3-year training program may be considered short when compared to the 5-year European standard [16–18]. When PSA by EPs was implemented, it usually commenced with adult PSA and then slowly progressed to paediatric PSA. However, in some of these pioneering EDs, paediatric PSA was restricted to be performed by anaesthesiologists and/or paediatricians, making it more difficult for these EPs to obtain sufficient training and exposure. Furthermore, countries where paediatric EM is a recognised sub-specialty of EM offer specific training during or after completion of the EM residency program. For example, in the UK, the training consists of 6 months in a paediatric ED approved for sub-specialty training and 6 months of ward-based paediatrics of which 3 months should be in the care of unconscious and critically ill children, such as in a paediatric ICU. Secondary to both low-volume EDs in the Netherlands (15,000–50,000 patients/year) and excellent general practitioner (GP) care, there is insufficient exposure to seriously ill paediatric patients in a 3-year time frame. This lack of exposure is further intensified by the strict Dutch Labour Law, which limits working hours up to a maximum of 48 per week including education hours [18]. Extending the training program in general or adding a PSA-fellowship to the training program with specific targets on both adult and paediatric PSA skills could aid the next generation of EPs. To close this gap, PSA training is since 2014 mandatory for all residency training programs in the country. Although this implementation may have a positive influence, it is not specified in the current curriculum that these PSA skills should be acquired to the same extent for both adult and paediatric patients. In addition, we believe not all EP mentors are currently competent to train the new generation of EPs because their training was before 2014. Before 2014, EM residency-training programs did not have a uniform approach in teaching PSA. Therefore, simultaneous implementation of mandatory vocational paediatric PSA training and credentialing like with annual online courses and practical recertification classes will be essential to achieve competency of paediatric PSA by all EPs.

We acknowledge that there are several limitations of this study. The response rate of 41.3% makes this a limited sample. However, the respondents represented 84.6% (55/65) hospitals with EPs on their staff. Therefore, we think our results are generally representative for PSA activities throughout the country. Since this study was aimed at EPs, we cannot draw any conclusions about PSA performance and guideline adherence in EDs without EPs. As with every survey, there is a significant risk for responder bias. We aimed to address this bias by guaranteeing anonymity and confidentiality of all individual survey data.

## Conclusions

This nationwide survey demonstrates that there is still a significant gap between the performance of adult and paediatric PSA in the ED even though PSA-related training, guideline adherence, and registration of PSA-related adverse events appear to be adequate. Enhancement of paediatric PSA training during and after the residency program in combination with an increase of EP-staff can help improve the availability of both adult and paediatric PSA in the ED. This may further improve the overall quality of ED-pain management.

## Additional file

Additional file 1: Procedural sedation survey for Dutch emergency physicians. (PDF 102 kb)

## Abbreviation

EM: Emergency department
EP: Emergency physicians
PSA: procedural sedation and analgesia
