# Supplementary material for: Procedural sedation and analgesia practices by emergency physicians in the Netherlands: a nationwide survey
Source: Int J Emerg Med. 2017 Dec 15;10:33. doi: 10.1186/s12245-017-0159-2 (PMC5732116; doi:10.1186/s12245-017-0159-2)
Supplement: Additional file 1: — Procedural sedation survey for Dutch emergency physicians. (PDF 102 kb) [file 12245_2017_159_MOESM1_ESM.pdf]

## Procedural Sedation Survey for Dutch Emergency Physicians - June 2016

**Demographic/general questions:**

1. In which hospital do you practice? (free text)

2. Is this a hospital with multiple locations? (yes/no)
- if Yes:
    - Do you practice emergency medicine at all locations?
    - Is Procedural sedation and Analgesia (PSA) provided in the Emergency Department (ED) at all locations

3. This hospital is:
    - An Academic hospital with a residency training program for Emergency Physicians (EPs)?
    - An Academic hospital without a residency training program for Emergency Physicians (EPs)?
    - A non-academic hospital with a residency training program for Emergency Physicians (EPs)?
    - A non-academic hospital with a residency training program for Emergency Physicians (EPs)?

4. What is your age? (dropdown)

5. Are you male/female?

6. In which country did you do your residency training (The Netherlands, Germany, Belgium, Other)

7. How many years have you been registered as "SEH-arts KNMG" with the RGS (*certified Emergency Physician*). In case you were registered before 2009, include years registered by the SOSG.

8. How many years have you practiced in you current hospital? (dropdown)

9. How many hours of clinical work do you perform in the Emergency Department (drop down).

10. Since what year have EPs been practicing in your hospital? (dropdown)

11. Is there 24/7 coverage by EPs in your ED? (yes/no)

12. How is the FTE of EPs calculated?
    - AMS
    - FWG
    - Don't know

If AMS: Of how many FTE AMS does the group of EPs in your hospital consist? (open)
If FWG: Of how many FTE FWG does the group of EPs in your hospital consist? (open)

13. Are the EPs represented in your hospital sedation committee? (yes/no)

**<u>PSA training:</u>**

**The following questions concern your PSA training <u>DURING</u> your Emergency Medicine residency-training program.**

14. Have you been trained in performing PSA in adult patients? (yes/no)
If yes: in which way:
- Only theoretical
- Only practical
- Both theoretical and practical

Was this sufficient: (yes/no)
If no, what were the reason(s) (multiple answers possible)
- Lack of exposure due to a short EM-training program
- Lack of exposure due to competition with other specialities
- Insufficient training because mentors did not perform PSA
- Insufficient airway management skill training
- Other: (free text)

15. Have you been trained in performing PSA in paediatric patients? (yes/no)
If yes: in which way:
- Only theoretical
- Only practical
- Both theoretical and practical

Was this sufficient: (yes/no)
If no, what were the reason(s) (multiple answers possible)
- Lack of exposure due to a short EM-training program
- Lack of exposure due to competition with other specialities
- Insufficient training because mentors did not perform PSA
- Insufficient airway management skill training

Other: (free text)

16. In 2012 the CBO guideline on PSA was published. Did you learn to perform PSA in accordance with this guideline during your residency? (yes/no)
If no, why not? (multiple answers possible)
- I completed my training before 2012
- My hospital did not use this guideline
- Other (free text)

**De following questions concerning PSA training <u>AFTER</u> the completion of your EM residency-training program.**

17. Which PSA training/course did you perform? (multiple answers possible)
- PSA course provided by the Netherlands Society of Emergency Physicians (NSEP)
- PSA course provided by a commercial /private company
- I was trained by my colleagues in the ED
    - Emergency Physician

- Anaesthesiologist
- Intensive Care specialist
- Paediatrician
- Surgeon
- Other (free texts)

- None

18. Do you need (more) training in PSA and or related skills? (yes/no)
If yes, which training would you want to do: multiple answers possible

- A PSA training course specifically for adult emergency patients
- A PSA training course specifically for paediatric emergency patients
- A general refresher course on PSA
- A fellowship in PSA in another hospital/ED where they do many PSA
- On site training in your ED by experienced physicians form another ED
- A specific airway management course for EPs
- Other: (free text)

**PSA in practice:**

19. Is each PSA procedure registered? (yes/no)
If yes, where is it registered (multiple answers possible)

- On the NSEP registration form
- On a local registration form
- In the electronic "Chipsoft" PSA registration form
- In the electronic patient file

20. Does your ED keep a record of all PSA related complications? (yes/no)
If yes:

- This is done by EPs as well as other specialists performing PSA in the ED (yes/no)
- This is done only by EPs (yes/no)

21. Do you know the content of the CBO guideline " PSA outside the operating room" (yes/no)
If yes:

- Do you use the CBO guideline on PSA in your current practice? (yes/no)
- Do you believe the CBO guideline to be user-friendly (yes/no)

If no: do you use another local protocol? (yes/no)

22. Do you know the content of the NSEP practical guideline on PSA for EPs? (yes/no)
If yes:

- Do you use it your current practice? (yes/no)
- Do you believe it to be user-friendly (yes/no)

If no: do you use another local protocol? (yes/no)

23. Do you know the content of the NSEP pocket card on PSA for EPs? (yes/no)
If yes:

- Do you use it your current practice? (yes/no)

- Do you believe it to be user-friendly (yes/no)

If no: do you use another local pocket card? (yes/no)

**PSA in adult patients:**

24. Do you perform PSA in adult patients (yes/no)

If yes:

- Since when do perform PSA in adult patients (year)
- How often do you perform PSA in adults on average?
  - 5 x a week
  - 2-4 x a week
  - 1 x per week
  - 1x every 2 weeks
  - 1x per month
  - < 1 x per month
- Is this frequency enough to maintain you skills? (yes/no)

If no, what is the reason you do not perform enough PSA in adults? (multiple answers possible)

- There are not enough adult patients with an indication for PSA
- PSA is standardly done by other specialties, namely:
  - Anaesthesiology
  - Surgery
  - Other (free text)
- Other specialities prohibit the provision of PSA by EPS, it is prohibited by:
  - Anaesthesiology
  - Surgery
  - Other (free text)
- There is lack of support by the Emergency nurses
- There are not enough EPs to cover other patient care during the PSA procedure
- We do not have a suitable space to perform PSA in our ED
- Other (free text)

25. Which sedative is your first choice when performing PSA in adult patients in the ED?

  - Propofol
  - Midazolam
  - Ketanest
  - Etomidate
  - $N_2O$ (50:50)
  - Other (free text)

26. Which analgesic medication is your first choice when performing PSA in adult patients in the ED?

  - Fentanyl
  - Sufentanil
  - Remifentanil
  - Morfine
  - Paracetamol
  - NSAID
  - Ketanest

- $N_2O$ 50:50
- Local analgesia (a.i. lidocaïne)
- None
- Other (free text)

27. Do you sometimes use the intra-nasal route for giving sedatives or analgesics in adult patients? (yes/no)
If yes, which medication do you provide intra-nasally? (multiple answers possible)

- Midalzolam
- Ketanest
- Fentanyl/Instanyl
- Other (free text)

28. What is the most common indication to perform PSA in adults in your ED?

- Dislocation of the hip
- Dislocation of the shoulder
- Dislocation of the elbow
- Other joint dislocation
- Dislocated fracture of the arm
- Dislocated fracture of the leg
- Incision & drainage of an abscess
- Cardioversion
- Foreign body removal
- Wound management face
- Wound management other
- CT scan
- Chest tube placement
- Other

29. Do you think all adult patients with an indication for PSA in your ED actually receive it (24/7)? (yes/no)
If no, what is/are the reason(s): (multiple answers possible)

- The ED is not staffed with EPS 24/7
- Despite 24/7 staffing with EPs, the EP is not always available
- Not all EPS in my ED are capable in performing PSA

Other (free text)

30. Who performs **most** of the adult PSA in your ED?

- Emergency Physician
- EP in training
- EP not in formal training
- Anaesthesiologist
- Sedation specialist ( nurse practitioner)
- Surgeon
- Cardiologist
- Intensive care specialist
- Orthopaedic surgeon

- Other specialist
- Anaesthesiologist in training
- Intensive care physician in training
- (Orthopaedic) surgeon in training
- Cardiologist in training

31. Who else performs adult PSA in your ED? (multiple answers possible)
    - Emergency Physician
    - EP in training
    - EP not in formal training
    - Anaesthesiologist
    - Sedation specialist (nurse practitioner)
    - Surgeon
    - Cardiologist
    - Intensive care specialist
    - Orthopaedic surgeon
    - Other specialist
    - Anaesthesiologist in training
    - Intensive care physician in training
    - (Orthopaedic) surgeon in training
    - Cardiologist in training

32. ***The following statements you can score 1-5.***
***1 = strongly disagree, 2= disagree, 3=neutral, 4= agree, 5= strongly agree***

- I am capable and competent in performing PSA in adults
- I am able to perform ALS appropriately
- I am aware of the (contra-)indications and precautions for PSA
- I am able to recognize and treat the most common adverse events and/or complications

**<u>PSA in paediatric patients</u>**

33. Do you perform PSA in paediatric patients (yes/no)
If yes:
- Since when do perform PSA in paediatric patients (year)
- How often do you perform PSA in paediatric patients on average?
  - 5 x a week
  - 2-4 x a week
  - 1 x per week
  - 1x every 2 weeks
  - 1x per month
  - < 1 x per month
- Is this frequency enough to maintain you skills? (yes/no)

If no, what is the reason you do not perform enough PSA in adults? (multiple answers possible)

- There are not enough paediatric patients with an indication for PSA
- PSA is standardly done by other specialties, namely:
  - Anaesthesiology
  - Paediatrics
  - Surgery
  - Other (free text)
- Other specialities prohibit the provision of paediatric PSA by EPS, it is prohibited by:
  - Anaesthesiology
  - Paediatrics
  - Surgery
  - Other (free text)
- There is lack of support by the Emergency nurses
- There are not enough EPs to cover other patient care during the PSA procedure
- We do not have a suitable space to perform paediatric PSA in our ED
- Other (free text)

34. Which sedative is your first choice when performing PSA in paediatric patients in the ED?

- Propofol
- Midazolam
- Ketanest
- Etomidate
- $N_2O$ (50:50)
- Other (free text)

35. Which analgesic medication is your first choice when performing PSA in paediatric patients in the ED?

- Fentanyl
- Sufentanil
- Remifentanil
- Morfine
- Paracetamol
- NSAID
- Ketanest
- $N_2O$ 50:50
- Local analgesia (a.i. lidocaïne)
- None
- Other (free text)

36. Do you sometimes use the intra-nasal route for giving sedatives or analgesics in paediatric patients? (yes/no)
If yes, which medication do you provide intra-nasally? (multiple answers possible)

- Midalzolam
- Ketanest
- Fentanyl/Instanyl
- Other (free text)

37. What is the most common indication to perform PSA in paediatric patients in your ED?

- Dislocation of the hip
- Dislocation of the shoulder
- Dislocation of the elbow
- Other joint dislocation
- Dislocated fracture of the arm
- Dislocated fracture of the leg
- Incision & drainage of an abscess
- Cardioversion
- Foreign body removal
- Wound management face
- Wound management other
- CT scan
- Chest tube placement
- Other

38. Do you think all paediatric patients with an indication for PSA in your ED actually receive it (24/7)? (yes/no)
If no, what is/are the reason(s): (multiple answers possible)

- The ED is not staffed with EPS 24/7
- Despite 24/7 staffing with EPs, the EP is not always available
- Not all EPS in my ED are capable in performing paediatric PSA
- Other (free text)

39. Who performs **most** of the paediatric PSA in your ED?

- Emergency Physician
- EP in training
- EP not in formal training
- Anaesthesiologist
- Sedation specialist (nurse practitioner)
- Surgeon
- Cardiologist
- Paediatrician
- Paediatric intensivist
- Intensive care specialist
- Orthopaedic surgeon
- Other specialist
- Anaesthesiologist in training
- Intensive care physician in training
- (Orthopaedic) surgeon in training
- Cardiologist in training

40. Who else performs paediatric PSA in your ED? (multiple answers possible)

- Emergency Physician

- EP in training
- EP not in formal training
- Anaesthesiologist
- Sedation specialist ( nurse practitioner)
- Surgeon
- Cardiologist
- Paediatrician
- Paediatric intensivist
- Intensive care specialist
- Orthopaedic surgeon
- Other specialist
- Anaesthesiologist in training
- Intensive care physician in training
- (Orthopaedic) surgeon in training
- Cardiologist in training

***41. The following statements you can score 1-5.***
***1 = strongly disagree, 2= disagree, 3=neutral, 4= agree, 5= strongly agree***

- I am capable and competent in performing PSA in paediatric patients
- I am capable to perform APLS appropriately
- I am aware of the (contra-)indications and precautions for paediatric PSA
- I am capable to recognize and treat the most common adverse events and/or complications
- I am capable to inform paediatric patients and/or its parents/legal guardians about PSA its potential adverse events and alternatives?
- I can guarantee child-friendly circumstances before, during and directly after the PSA
- I can provide topical analgesia when appropriate
- The age of the child determines if I perform PSA or not
- I only provide PSA to paediatric patients in the direct presence/ under direct supervision of another specialist
- I only provide paediatric PSA after consulting an anaesthesiologist/paediatrician
